# Beyond preserving Roe: Honoring Ruth Bader Ginsburg by promoting gender equity and reproductive justice

**DOI:** 10.1016/j.eclinm.2020.100595

**Published:** 2020-10-15

**Authors:** Sarah Averbach

**Affiliations:** a Division of Family Planning, Department of Obstetrics, Gynecology and Reproductive Sciences, University of California, San Diego, CA, United States; b Center on Gender Equity and Health, Division of Infectious Disease and Global Public Health, University of California, San Diego, CA, United States

“The decision whether or not to bear a child is central to a woman's life, to her wellbeing and dignity.”

Ruth Bader Ginsburg, 1993

A 1973 article in the New England Journal of Medicine describes a 23-year-old pregnant mother of two who died after her husband found her in their bathroom hemorrhaging with a puncture wound in her lower abdomen [1]. Tragedies of this sort are now rare in the United States (U.S.) despite the fact that abortion remains common – 1 in 4 women will have an abortion by age 45 [2]. The fact that abortion is safe and legal in the U.S. is due in large part to the landmark 1973 Supreme Court case, Roe v. Wade, which legalized abortion nationwide. Before Roe, however, it's estimated that more than 1000 women died each year in the U.S. from unsafe abortion [3]. In comparison, in 2015, the last year data are available, only 2 confirmed abortion-related deaths were reported [4].

For years, opponents have attempted to erode access to abortion through restrictive legislation passed under the pretext of ensuring the safety of abortion. For example, TRAP laws (targeted regulation of abortion providers) create medically unnecessary regulations and standards for abortion care, and the Food and Drug Administration requires that medication abortion be dispensed only in health-care settings by certified prescribers.

Justice Ruth Bader Ginsburg's recent death amplifies how tenuous abortion access is in the U.S.. President Trump has made clear he intends to appoint anti-choice Supreme Court judges who will overturn Roe. If the Republicans hastily appoint another conservative judge to the Court to replace Ginsburg weeks before the election, it could re-shape the Court for years to come. There will be a majority of judges on the court who are willing to overturn Roe despite the fact that most Americans oppose overturning the landmark case [5]. The preponderance of evidence suggests that **laws criminalizing abortions do not change their prevalence but rather decrease their safety.** Worldwide, in countries where abortion is prohibited, or only permitted to save a woman's life, abortion rates are similar to countries where abortion is available on request, [6] but death from unsafe abortion becomes one of the leading causes of maternal mortality in countries lacking access [7]. President Trump's efforts to overturn Roe sacrifice women's safety and reproductive autonomy for political gain.

**Access to abortion and contraception are the cornerstone of gender equity.** Access to comprehensive reproductive healthcare, that includes prenatal care, contraception and abortion, is fundamental to women's economic empowerment. The ability to prevent or delay childbirth allows women to pursue educational attainment and career development. In the U.S., women denied a wanted abortion are more likely to live in poverty and less likely to be employed than women who receive a desired abortion [8]. Ruth Bader Ginsburg was a consummate advocate for gender equity. Although Roe v. Wade supports abortion access based on the right to privacy, Ginsburg proposed that our laws should go further to protect a woman's right to bodily autonomy. “*If you impose restraints that impede her choice, you are disadvantaging her because of her sex,”* she told the Senate.

**If Roe is overturned, the right to abortion will fall to states.** Abortion will remain legal in states that haven enshrined this right in their laws— such as New York and California. However, **women who live in restrictive states will be forced to travel for abortion care, undergo illegal abortions or continue unwanted pregnancies**. Southern states have some of the most restrictive abortion laws and also higher proportions of racial minorities and poverty. Due to structural racism, women of color in the U.S. experience income inequality and worse maternal and reproductive health outcomes including higher rates of unintended pregnancy [9,10]. Therefore, **if Roe is overturned it will disproportionately harm poor women and women of color.**

*Reproductive Justice* is described by *SisterSong*, the Women of Color Reproductive Justice Collective, as “the human right to maintain personal bodily autonomy, have children, not have children and parent the children we have in safe and sustainable communities.” We must preserve Roe, but **preserving Roe is not enough**. Even when abortion is legal, many women, particularly women of color, cannot afford to safely travel to the nearest clinic or pay out of pocket for an abortion. We must fight for laws that not only protect abortion access but promote Reproductive Justice.

If we look to our country's history, and to other countries where abortion is currently restricted, it is clear that if Roe is overturned it will deeply erode efforts to achieve gender equity and Reproductive Justice– disproportionately harming poor women and women of color. As physicians and scientists, we must do more than fight to preserve the legal precedent of Roe— we must honor Ruth Bader Ginsberg's legacy and advocate for more durable federal legislation that protects reproductive autonomy.

*The views presented are the author's and do not necessarily represent those of the journal or the institution.*

## Declaration of Competing Interest

Dr. Averbach reports nothing to disclose.
